# Synergistic effect of myocardial injury and mid-regional proAdrenomedullin elevation in determining clinical outcomes of SARS-CoV-2 patients

**DOI:** 10.3389/fmed.2022.929408

**Published:** 2022-10-26

**Authors:** Silvia Spoto, Fabio Mangiacapra, Giorgio D’Avanzo, Daniela Lemme, César Bustos Guillén, Antonio Abbate, John Daniel Markley, Federica Sambuco, Roshanak Markley, Marta Fogolari, Luciana Locorriere, Domenica Marika Lupoi, Giulia Battifoglia, Sebastiano Costantino, Massimo Ciccozzi, Silvia Angeletti

**Affiliations:** ^1^Department of Diagnostic and Therapeutic Medicine, University Campus Bio-Medico of Rome, Rome, Italy; ^2^Unit of Cardiovascular Science, University Campus Bio-Medico of Rome, Rome, Italy; ^3^Division of Infectious Diseases, Department of Internal Medicine, Clinica Universidad de los Andes, Santiago Metropolitan, Chile; ^4^Division of Cardiology, Department of Internal Medicine, Pauley Heart Center, Virginia Commonwealth University, Richmond, VA, United States; ^5^Division of Infectious Disease and Epidemiology, Department of Internal Medicine, Virginia Commonwealth University, Richmond, VA, United States; ^6^Central Virginia, Veterans Administration Hospital, Richmond, VA, United States; ^7^Department of Emergency, University Campus Bio-Medico of Rome, Rome, Italy; ^8^Unit of Clinical Laboratory Science, University Campus Bio-Medico of Rome, Rome, Italy; ^9^Labotarory Research Unit, Fondazione Policlinico Universitario Campus Bio-Medico, Rome, Italy; ^10^Unit of Medical Statistics and Molecular Epidemiology, University Campus Bio-Medico of Rome, Rome, Italy

**Keywords:** myocardial injury, mid-regional proAdrenomedullin, COVID-19, Troponin I (tni), SARS-CoV-2

## Abstract

**Objective:**

Coronavirus disease 2019 (COVID-19) is a systemic disease induced by SARS-CoV-2 causing myocardial injury. To date, there are few data on the correlation between mid-regional proAdrenomedullin (MR-proADM) and myocardial injury. The aim of this study was to evaluate whether the association of myocardial injury and elevated mid-regional proAdrenomedullin values could predict mortality of SARS-CoV-2 patients, to offer the best management to COVID-19 patients.

**Materials and methods:**

All patients hospitalized for SARS-CoV-2 infection at the COVID-19 Center of the Campus Bio-Medico of Rome University were included between October 2020 and March 2021 and were retrospectively analyzed. Myocardial injury was defined as rising and/or fall of cardiac hs Troponin I values with at least one value above the 99th percentile of the upper reference limit (≥15.6 ng/L in women and ≥34.2 ng/L in men). The primary outcome was 30-day mortality. Secondary outcomes were the comparison of MR-proADM, CRP, ferritin, and PCT as diagnostic and prognostic biomarkers of myocardial injury. Additionally, we analyzed the development of ARDS, the need for ICU transfer, and length of stay (LOS).

**Results:**

A total of 161 patients were included in this study. Of these, 58 (36.0%) presented myocardial injury at admission. An MR-proADM value ≥ 1.19 nmol/L was defined as the optimal cut-off to identify patients with myocardial injury (sensitivity 81.0% and specificity 73.5%). A total of 121 patients (75.2%) developed ARDS, which was significantly more frequent among patients with myocardial injury (86.2 vs. 68.9%, *p* = 0.015). The overall 30-day mortality was 21%. Patients with myocardial injury presented significantly higher mortality compared to those without the same (46.6 vs. 6.8%, *p* < 0.001). When dividing the entire study population into four groups, based on the presence of myocardial injury and MR-proADM values, those patients with both myocardial injury and MR-proADM ≥ 1.19 nmol/L presented the highest mortality (53.2%, *p* < 0.001). The combination of myocardial injury and MR-proADM values ≥ 1.19 nmol/L was an independent predictor of death (OR = 7.82, 95% CI = 2.87–21.30; *p* < 0.001).

**Conclusion:**

The study is focused on the correlation between myocardial injury and MR-proADM. Myocardial injury induced by SARS-CoV-2 is strongly associated with high MR-proADM values and mortality.

## Introduction

Coronavirus disease 2019 (COVID-19) is a systemic disease induced by Severe Acute Respiratory Distress Syndrome *Coronavirus* 2 (SARS-CoV-2) causing widespread endothelial damage primarily involving the pulmonary and cardiovascular systems (1–4).

Acute cardiac injury in COVID-19 patients is present in upto 15–50% of critically ill patients and is represented by myocardial injury, endothelitis, heart failure, Takotsubo cardiomyopathy, acute coronary syndromes, pulmonary thromboembolism, and arrhythmias (2, 5–8).

Myocardial injury is defined as an increase in myocardial enzyme levels (Troponin) with at least one value above the 99th percentile upper reference limit in absence of myocardial ischemia and can be caused by several mechanisms (9). Myocardial injury occurs due to indirect or direct myocardial damage with a mortality of 60% (8).

*Indirect myocardial injury* evidenced by the increase of Troponin is present in up to 36% in the early course of SARS-CoV-2 infection and it is associated with an increased risk of requiring mechanical ventilation, fatal ventricular arrhythmias, and a 59.6% of risk mortality (10–15).

*A direct myocardial injury* affects hs Troponin I in case of acute coronary syndrome and could affect adrenomedullin expression that is expressed by cardiomyocytes, pericytes, cardiofibroblasts, endothelial cells, epicardial adipose cells, vascular endothelial cells, smooth muscle cells, and migratory angiogenic cells (16, 17).

Currently, the understanding of the underlying physiopathological mechanisms of the onset of myocardial injury is still limited and there are only little data on the correlation between myocardial injury and MR-proADM. This biomarker helps clinicians in identifying those patients with severe disease and at higher risk of death (4, 18–22).

The aim of this study was to evaluate whether the association of myocardial injury and elevated mid-regional proAdrenomedullin values could predict mortality of SARS-CoV-2 patients, to offer the best management to COVID-19 patients.

## Materials and methods

### Patient selection and characteristics

All patients hospitalized with SARS-CoV-2 infection at the COVID-19 Center of the Campus Bio-Medico of Rome University were included between October 2020 and March 2021 and were retrospectively analyzed. The COVID-19 Center includes the Medicine Department with a sub-intensive care unit.

We included all patients with a positive reverse transcription polymerase chain reaction test (RT-PCR) for SARS-CoV-2, with hs Troponin I and MR-proADM assessment. We excluded patients < 18 years old and pregnant women.

The study was approved by the Ethical Committee of the University Campus Bio-Medico of Rome.

All methods were performed in accordance with the relevant guidelines and regulations available at that moment.

The control group consisted of patients with SARS-CoV-2 infection without increased hs Troponin I or acute coronary syndrome (ACS), pericarditis, or myocarditis admitted to the COVID-19 center in the same period.

### Clinical outcomes and definitions

Primary outcome of the study was 30-day mortality. Secondary outcomes were the comparison of MR-proADM, CRP, ferritin, and PCT as diagnostic and prognostic biomarkers of myocardial injury. Additionally, we analyzed the development of ARDS, the need for ICU transfer, and length of stay (LOS).

Myocardial injury was defined by the rise and/or fall of cardiac hs Troponin I values with at least one value above the 99th percentile of the upper reference limit; ARDS was defined according to the Berlin definition (9, 10, 23).

The following data were collected at inclusion: demographic characteristics (age and gender), onset symptoms, relevant comorbidities, immune status (active malignancy or other causes of immunosuppression), concomitant antimicrobial, use of antiretroviral medication, immunosuppressive treatments, and clinical presentation.

All patients received a complete physical examination including body temperature, blood pressure, heart and respiratory rate, cardiac, pulmonary, abdominal, and neurological evaluation, electrocardiogram, and chest tomography, while an echocardiogram was performed only if clinically needed.

Laboratory tests performed at inclusion were complete blood count (CBC), hs Troponin I, MR-proADM, CRP, ferritin, PCT, D-Dimer, INR, TTPA, liver function test, creatinine, arterial blood gases, and serum lactate.

All patients received standard of care based on disease severity and need for oxygen support. When needed, patients received low-molecular weight heparin, remdesivir, and steroid therapy.

All included patients were followed until death or 30-day follow-up, whichever came first.

### Laboratory markers

Diagnosis of COVID-19 was performed by molecular testing through RT-PCR on a nasopharyngeal swab and/or endotracheal aspirate, detecting spike three SARS-CoV-2 genes (S, N, and E or S, RdRP, and N genes) (4).

Myocardial injury was considered when hs Troponin I was ≥15.6 ng/L in women and ≥34.2 ng/L in men.

Ferritin, hs Troponin I, and CRP were measured by Alinity c (Abbott, diagnostics) following the manufacturer’s instruction. Normal ranges are shown in Table 1. CBC was performed on a whole blood sample by Sysmex XE-9000 (Dasit, Italy) following the manufacturer’s instruction. NLR was calculated by the ratio between absolute values of neutrophils and lymphocytes. MR-proADM and PCT plasma concentrations were measured on an automated Kryptor analyzer, using a time-resolved amplified cryptate emission (TRACE) technology assay (Kryptor PCT; Brahms AG; Hennigsdorf, Germany), with commercially available immunoluminometric assays (Brahms) (24–27).

**TABLE 1:** Normal range and methodology for biomarkers assessment.

Biomarker	Sample	Methodology	Sex	Normal range values	Unit
hs Troponin I	Plasma	Chemiluminescence	Female	0–15.6	pg/mL
hs Troponin I	Plasma	Chemiluminescence	Male	0–34.2	pg/mL
MR-proAdrenomedullin	Plasma	TRACE* assay		0.00–0.50	nmol/L
C-reactive protein	Plasma	Turbidimetric	Female/Male	≤0.5	mg/dL
Ferritin	Serum	Chemiluminescence	Female	4.63–204	ng/mL
Ferritin	Serum	Chemiluminescence	Male	21.81–264.66	ng/mL
Procalcitonin	Plasma	TRACE* assay		0.00–0.50	ng/mL
Neutrophils-absolute value	Whole blood	Flow cytometric		2.00–7.00	×10^3^/μL
Lymphocytes-absolute value	Whole blood	Flow cytometric		1.00–3.00	×10^3^/μL

*TRACE, time-resolved amplified cryptate emission technology assay.

### Statistical analysis

As appropriate, continuous variables were reported as mean (standard deviation) or as median (interquartile range). Categorical variables were reported as frequencies and percentages. Comparisons between continuous variables were performed using Student’s *t*-test or the Mann-Whitney *U*-test. Comparison between categorical variables was evaluated using the Fisher exact test or the Pearson chi-square test, as appropriate. The normal distribution of continuous variables was tested with the Shapiro-Wilk test. Correlation between continuous variables was assessed using the Spearman rank test. A receiver operating characteristic (ROC) curve analysis was used to test the ability of laboratory values to discriminate between patients with and without myocardial injury and patients who died and did not during the hospital stay. The optimal cutoff point was calculated by determining the value that provided the greatest sum of sensitivity and specificity. All baseline clinical features were evaluated in univariate analysis for the association with myocardial injury and death using logistic regression. Only variables with a *p*-value < 0.05 were considered for the final multivariable logistic regression models, providing odds ratios (ORs) and 95% confidence intervals (CI). Statistical analysis was performed using Stata/IC version 14.0 (STATA Corp., College Station, TX, USA), and *p*-values < 0.05 (2-tailed) were considered significant.

## Results

### Study population

A total of 161 patients were included in this study. Of these, 58/161 (36%) presented myocardial injury at admission. The characteristics of the patients are shown in Table 2. A total of 99/161 (61.5%) patients were males. Among them, 32/99 (32%) developed myocardial injury (*p* = 0.21).

**TABLE 2:** Characteristics of patients.

Variable	Overall population (*n* = 161)	Myocardial injury (*n* = 58)	Absence of myocardial injury (*n* = 103)	*P-value*
Age [years (IQR)]	73 (62–81)	79 (73–83)	67 (58–80)	<0.001
Male sex [*n* (%)]	99 (61.5)	32 (55.2)	67 (65.0)	0.216
**Cardiovascular risk factors [*n* (%)]**				
Diabetes mellitus	48 (29.8)	20 (34.4)	28 (27.2)	0.331
Hypertension	106 (65.8)	44 (75.9)	62 (60.2)	0.044
Dyslipidemia	61 (37.9)	26 (44.8)	35 (34.0)	0.173
Smoking habit	26 (16.1)	12 (20.6)	14 (13.6)	0.240
BMI > 30 kg/m^2^	25 (15.5)	13 (22.4)	12 (11.7)	0.070
Coronary artery disease [*n* (%)]	34 (21.1)	18 (31.0)	16 (15.5)	0.021
Chronic pulmonary disease [*n* (%)]	30 (18.6)	16 (27.6)	14 (13.6)	0.029
Chronic kidney disease [*n* (%)]	27 (16.8)	16 (27.9)	11 (10.7)	0.006
Chronic liver disease [*n* (%)]	9 (5.6)	2 (3.4)	7 (6.8)	0.375
Active cancer [*n* (%)]	24 (14.9)	9 (15.5)	15 (14.6)	0.870
**Laboratory [median (IQR)]**				
hs Troponin I [ng/l]	11 (10–49)	83 (42–226)	10 (10–10)	<0.001
MR-proADM [nmol/l]	1.12 (0.78–1.91)	1.90 (1.24–3.83)	0.88 (0.66–1.29)	<0.001
CRP [mg/dl]	6.6 (2.3–11.9)	10.9 (6.8–15.7)	3.6 (1.4–8.0)	<0.001
Ferritin [ng/ml]	802 (279–1540)	1403 (621–2230)	635 (247–1299)	<0.001
PCT [ng/ml]	0.07 (0.04–0.37)	0.21 (0.07–0.83)	0.06 (0.03–0.10)	<0.001
Leukocytes [unit/μl]	9800 (7120–12300)	12275 (8440–16040)	9080 (6470–11420)	<0.001
Neutrophils [unit/μl]	8230 (5280–10920)	10275 (7110–13960)	6910 (4510–9490)	<0.001
Lymphocytes [unit/μl]	930 (560–1460)	765 (430–1110)	1020 (610–1560)	0.013
Neutrophil/Lymphocyte ratio	9.68 (4.22–15.58)	12.97 (7.00–26.50)	7.98 (3.33–13.07)	<0.001
PaO2/FiO2	216 (108–327)	145 (85–281)	252 (130–357)	<0.001
ICU admission [*n* (%)]	41 (25.5)	17 (29.3)	24 (23.3)	0.401
Median hospital stay [days (IQR)]	14 (7–23)	15 (8–26)	12 (7–21)	0.387
ARDS [*n* (%)]	121 (75.2)	50 (86.2)	71 (68.9)	0.015

Patients with myocardial injury were significantly older (79 [IQR = 73–83] vs. 67 [IQR = 58–80] years old, *p* < 0.001) than those without myocardial injury. These patients had a more frequent history of Hypertension (75.9 vs. 60.2%, *p* = 0.044), coronary artery disease (31 vs. 15.5%, *p* = 0.021), chronic pulmonary disease (27.6 vs. 13.6%, *p* = 0.029), and chronic kidney disease (27.9 vs. 10.7%, *p* = 0.006). Among the patients with myocardial injury, 3/58 (5.17%) had acute coronary syndrome.

In the overall population, 41/161 patients (25.5%) were admitted to the intensive care unit during the hospitalization, and the median hospital stay was 14 (7–23 IQR) days. A total of 121/161 patients (75.2%) developed ARDS, which was significantly more frequent among patients with myocardial injury (86.2 vs. 68.9%, *p* = 0.015). In-hospital mortality was 21% (34/161 of the overall population) and 46.6% (27/58 of patients with myocardial injury).

### Myocardial injury

A significant correlation was found between hs Troponin I and MR-proADM levels (Spearman *r* = 0.569, *p* < 0.001). An MR-proADM value ≥ 1.19 nmol/L was defined as the optimal cut-off to identify patients with myocardial injury. This cut-off had 81.0% of sensitivity and 73.5% of specificity.

**FIGURE 1:**
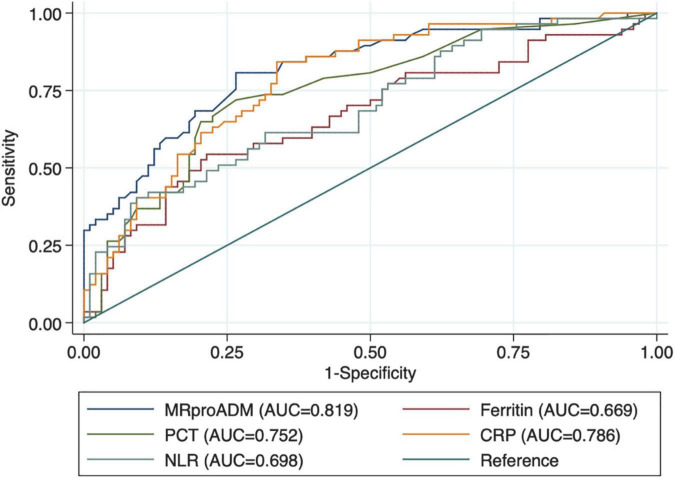
Receiver operating characteristic curves (ROC) of biomarkers in patients with myocardial injury.

As reported in Table 2, patients with myocardial injury at admission showed significantly higher values of MR-proADM, CRP, ferritin, PCT, and neutrophil/lymphocyte ratio. At ROC curve analysis, all laboratory markers were able to discriminate between patients with and without myocardial injury (Figure 1 and Table 3). However, MR-proADM showed the greatest area under the curve ([AUC] 0.818, 95% CI = 0.750–0.875; *p* < 0.001). Pairwise comparison showed that the AUC of MR-proADM was significantly greater than the AUC of ferritin (*p* = 0.010) and neutrophil/lymphocyte ratio (0.021), but similar to that of CRP and PCT.

**TABLE 3:** Receiver operating characteristic (ROC) curves of laboratory markers for myocardial damage and pairwise comparison between mid-regional proAdrenomedullin (MR-proADM), C-reactive protein, ferritin, procalcitonin, and neutrophil/lymphocyte ratio.

	AUC	95% CI	*P-value* (vs MR-proADM)	Optimal cut-off
MR-proADM	0.818	0.750–0.875	–	1.19 nmol/L
CRP	0.786	0.713–0.858	0.386	5.67 mg/dL
Ferritin	0.669	0.578–0.761	0.010	1,403 ng/mL
PCT	0.752	0.672–0.832	0.131	0.1 ng/mL
Neutrophil/Lymphocyte ratio	0.698	0.611–0.783	0.021	12.67

### Predictors of myocardial injury

At univariate analysis (Table 4), age, hypertension, a history of coronary artery disease, chronic pulmonary disease, chronic kidney disease, and MR-proADM ≥ 1.19 nmol/L were significantly associated with an increased risk of myocardial injury. In the multivariate analysis (Table 4), an MR-proADM value of ≥1.19 nmol/L was an independent predictor of increased risk of myocardial injury (OR = 7.25, 95% CI = 2.93–17.9, *p* < 0.001).

**TABLE 4:** Logistic regression analysis for myocardial injury.

	Univariate analysis			Multivariate analysis		
		
	OR	95%CI	*P-value*	OR	95%CI	*P-value*
Age	1.06	1.03–1.10	<0.001	1.03	0.99–1.07	0.123
Hypertension	2.08	1.01–4.27	0.046	1.07	0.42–2.76	0.882
Coronary artery disease	2.45	1.13–5.29	0.023	1.18	0.45–3.10	0.743
Chronic kidney disease	3.19	1.36–7.45	0.008	1.28	0.47–3.51	0.631
Chronic pulmonary disease	2.42	1.08–5.42	0.031	1.54	0.56–4.26	0.400
MR-proADM ≥ 1.19 nmol/l	11.4	5.21–25.14	<0.001	7.25	2.93–17.9	<0.001

OR, odds ratio; CI, confidence interval.

### Predictors of death

Overall, 30-day death occurred in 34 (21.1%) patients and was significantly more frequent among those with myocardial injury (46.6 vs. 6.8%, *p* < 0.001). In the ROC curve analysis, MR-proADM was able to discriminate between patients who died and those who did not (AUC = 0.822, 95% CI = 0.751–0.877; *p* < 0.001; optimal cut-off ≥ 1.19 nmol/L). Among patients with MR-proADM values ≥ 1.19 nmol/L (*n* = 72), the incidence of death was significantly higher compared with those patients with low MR-proADM values (40.3 vs. 5.9%, *p* < 0.001). Also, when only considering patients with myocardial injury, MR-proADM was able to discriminate between patients who died and those who did not (AUC = 0.690, 95% CI = 0.551–0.820; *p* = 0.007; optimal cut-off ≥ 4.01 nmol/L). This cut-off had 40.7% of sensitivity and 89.7% of specificity.

**FIGURE 2:**
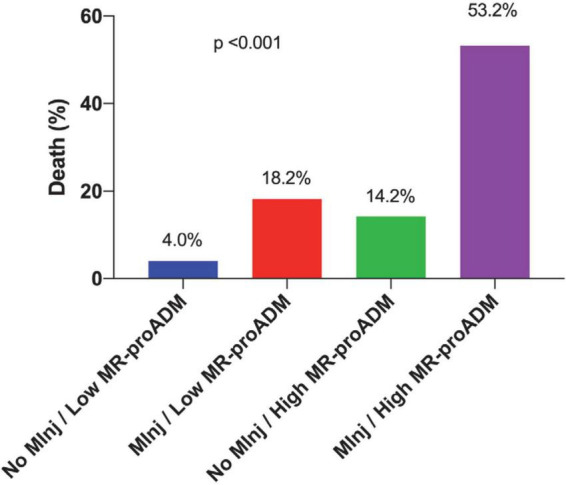
Incidence of in-hospital death according to the presence of myocardial injury and mid-regional proAdrenomedullin (MR-proADM) values of ≥1.19 nmol/L.

When dividing the entire study population in four groups based on the presence of myocardial injury and MR-proADM values, 75 patients (46.6%) had no myocardial injury and MR-proADM < 1.19 nmol/L, 11 patients (6.8%) had a myocardial injury and MR-proADM < 1.19 nmol/L, 28 patients (17.4%) had no myocardial injury and MR-proADM ≥ 1.19 nmol/L, and 47 patients (29.2%) had a myocardial injury and MR-proADM ≥ 1.19 nmol/L. Death occurred in 3/75 (4.0%), 2/11 (18.2%), 4/28 (14.2%), 25/47 (53.2%), respectively (*p* < 0.001; Figure 2).

In the univariate analysis (Table 5), age, ARDS, myocardial injury, MR-proADM values of ≥1.19 nmol/L, and the combination of myocardial injury and MR-proADM values of ≥1.19 nmol/L were significantly associated with an increased risk of death.

**TABLE 5:** Logistic regression analysis for death.

	Univariate analysis			Multivariate analysis			Multivariate analysis		
			
	OR	95%CI	*P-value*	OR	95%CI	*P-value*	OR	95%CI	*P-value*
Age	1.06	1.02–1.10	0.003	1.03	0.98–1.08	0.317	1.04	0.99–1.09	0.122
ARDS	14.93	1.97–113.21	0.009	10.58	1.26–88.95	0.030	10.74	1.27–91.10	0.029
Myocardial injury	12.50	4.93–31.68	<0.001	4.87	1.63–14.52	0.005			
MR-proADM ≥ 1.19 nmol/L	10.79	3.90–29.89	<0.001	3.39	0.96–12.02	0.058			
MInj/MR-proADM ≥ 1.19 nmol/L	14.31	5.82–35.18	<0.001				7.82	2.87–21.30	<0.001

OR, odds ratio; CI, confidence interval; MInj, myocardial Injury.

When myocardial injury and MR-proADM values of ≥1.19 nmol/L were entered separately in the same multivariate model, myocardial injury (OR = 4.87, 95% CI = 1.63–14.52, *p* = 0.005) and ARDS (OR = 10.58, 95% CI = 1.26–88.95, *p* = 0.030) were independent predictors of increased risk of death (Table 5). In a separate model, the combination of myocardial injury and MR-proADM values ≥ 1.19 nmol/L was an independent predictor of death with an OR of 7.82 (95% CI = 2.87–21.30, *p* < 0.001; Table 5).

## Discussion

The epidemiological data on myocardial injury in the literature is discordant. In the same way, the pathophysiological mechanism of myocardial injury onset is still unclear.

Consistent with the published data, 36% of the study population developed myocardial injury (11, 12). Among this group of patients, 27/58 (46.6%) died, in comparison with the 60% described in the literature (11, 12).

The mortality of patients with myocardial injury with both elevated values of hs Troponin I and MR-proADM ≥ 1.19 nmol/L reached 53.2% vs. mortality of 14.8% in the case of the elevated value of hs Troponin I only. Furthermore, the elevation of both biomarkers allowed the identification of patients with myocardial injury at higher mortality risk. In fact, if they were both negative the mortality was only 4%, but if both of them were positive, the mortality reached 53.2%.

These results agree with previous reports, where MR-proADM ≥ 2 nmol/L identified those patients affected by moderate/severe COVID-19 with high mortality risk related to multiple organ dysfunction syndrome, while values ≥3 nmol/L were predictive for ARDS development (4).

While an MR-proADM value of ≥1.19 nmol/L allows identifying patients with myocardial injury with high sensitivity and specificity, an MR-proADM value of ≥4.01 nmol/L allows identifying patients with myocardial injury at high risk of death with high specificity.

Therefore, the dosage of MR-proADM allows stratifying patients with myocardial injury at high risk of death by identifying patients who may also benefit from therapy with adrecizumab.

The median value of hs Troponin I in case of myocardial injury resulted in 83 vs. 11 ng/L of the overall population. Some studies had reported an optimal cut-off of 17 ng/L for Troponin T to predict mortality and of 0.03 μg/L for Troponin I in COVID-19 patients with cardiovascular disease (15, 28). These data suggest a role of hs Troponin I, not only as a marker of ischemia but also as a relevant biomarker of global stress for myocardial injury. In this way, hs Troponin I could be used to guide the prognosis and clinical management of the patients.

Our study shows that MR-proADM ≥ 1.19 nmol/L expresses myocardial injury with high diagnostic accuracy (sensitivity 81% and specificity 73.5%) when compared to ferritin and NLR ratio.

Of all bio-markers, MR-proADM was found to be the most specific of myocardial injury and SARS-CoV-2-related mortality.

MR-proADM ≥ 1.19 nmol/L has been shown to be an independent predictor of increased risk of myocardial injury and it has been significantly associated with risk factors of myocardial injury such as age, hypertension, history of coronary artery disease, and chronic pulmonary or kidney disease.

Age ≥ 65 years, male sex, and multicomorbidities increase the possibilities for developing severe SARS-CoV-2 infection, while pre-existing cardiovascular diseases, such as hypertension, diabetes mellitus, coronary artery disease, and heart failure, are associated with a worse prognosis (10, 14, 29–31).

Myocardial injury and MR-proADM ≥ 1.19 nmol/L were independent predictors of death (*p* < 0.001).

According to the literature, myocardial injury was also a predictor of in-hospital mortality.

Also, considering that acute cardiac injury in patients who died of COVID-19 has been reported in 35%, with detection of SARS-CoV-2 within the myocardium in 47% of post-mortem studied hearts (2, 5–7). Furthermore, one-third of severely ill COVID-19 patients develop acute kidney failure. Many of them require hemodyalitic procedures. This complication could weaken the diagnostic accuracy of Troponin value in the assessment of cardiac injury (10). It would be desirable to evaluate in further studies the combined dosage of hs Troponin I and MR-proADM, which could allow us to estimate with greater accuracy the real incidence of myocardial injury also in the absence of chest pain, troponin assessment, or evaluation of myocardial contractility.

The study has the limitation of being a single-center study and therefore the data obtained should be further confirmed by multicentric studies.

To our knowledge, this is one of the few studies that focused on the correlation between myocardial injury and MR-proADM. Values of MR-proADM ≥ 1.19 nmol/L correlate with myocardial injury and widespread endothelitis severity.

A myocardial injury might occur during SARS-CoV-2 infection as a consequence of myocardial, pulmonary, and endothelial damage. The mechanisms involved are represented by hypoxia that induces a decreased oxygen supply to the heart, causing modest or massive elevation of Troponin concentration, which is not necessarily correlated with deterioration of systolic left ventricular function but could be associated with right ventricular dysfunction due to acute right ventricular overload secondary to parenchymal or vascular lung disease resulting in subendocardial damage of the right ventricular myocardium in 19% of cases and by cytokine-induced injury (10, 15, 28, 32–35).

Adrenomedullin (ADM) is a protein that is released by endothelial and vascular smooth muscle cells following volume overload with the aim to preserve the endothelial barrier function. It binds to receptors prevalently found in cardiovascular and pulmonary systems (36–38). ADM induces vasodilatation, with consequent blood flow increase by reducing vasoconstriction acting as an inhibitor of the renin–angiotensin–aldosterone system (RAS). Furthermore, ADM contributes to endothelial integrity decreasing vascular permeability and acts as a bronchodilator.

Hypoxia, inflammatory cytokines, bacterial or viral products, shear stress, and vascular leakage represent stimuli for ADM up-regulation as it happens during SARS-CoV-2 infection, contributing to the failure of the ADM regulation (4, 39–41).

Disruption of the ADM system leads to (1) decrease of vascular resistance and capacitance vessels determining blood flow increase with hypoxic cardiac ischemia. (2) RAS activity reduces vasoconstriction, which leads to vascular leakage, increasing inflammation, and activation of the coagulation cascade. Additionally, RAS activation increases edema, oxidation, proliferation, and fibrosis, resulting in hypoxic cardiac ischemia and diffuse endothelitis that can lead to multiorgan failure (4, 42–50).

The mid-regional proAdrenomedullin (MR-proADM) is a peptide derived from ADM in a 1:1 ratio that can be used as a biomarker of organ failure, disease severity, and mortality in patients with COVID-19 (4, 51).

The alterations in endothelial cell lining are adaptive or maladaptive depending on disease extension, the time elapsed from disease onset, long-lasting viral shedding, and the host’s genetic heritage that expresses more or less ADM receptors, determining the extent of the immune-metabolic-inflammatory response. Instead, SARS-CoV-2 loads or variants have not so far indicated to influence the extent of organ damage (1, 52, 53).

Therefore, the role of ADM in COVID-19-related organ damage might suggest the use of new therapeutic agents, such as monoclonal antibodies. Adrecizumab, a humanized, monoclonal, non-neutralizing ADM-binding antibody could be used to improve vascular integrity, tissue congestion, and thereby clinical outcomes (18, 19).

Furthermore, the high incidence of myocardial injury caused by SARS-CoV-2 corresponds to that observed in other viral infections, such as Influenza, in which myocardial damage was detected as asymptomatic cardiac involvement in 0–53% of cases, with the presence of electrocardiogram alterations on roughly 50% of patients or highlighted post-mortem by the presence of myocarditis, pericarditis or acute coronary syndrome (14, 15, 54–58). Viral infections, indeed, can determine endothelial dysfunction up to apoptosis rousing coronary vasoconstriction and procoagulant state causing activation of plaque to hemodynamic instability (59).

Vaccination represents the best preventive method for both adults and children with effectiveness rates of 65–95 vs. 50–60% for Influenza, respectively, mostly in high-risk patients (>65 years, young children, presence of comorbidities, and immunocompromised patients), and it could be useful to prevent cardiovascular damage reducing mortality (59–65).

## Conclusion

Myocardial injury induced by SARS-CoV-2 is relevant.

The elevation of hs Troponin I and MR-proADM allows the identification of patients with myocardial injury at higher mortality risk.

An MR-proADM value of ≥1.19 nmol/L identifies patients with myocardial injury, and a MR-proADM value of ≥4.01 nmol/L identifies patients with myocardial injury at high risk of death.

Therefore, the dosage of MR-proADM allows stratifying patients with myocardial injury at high risk of death to offer the best management to critically ill COVID-19 patients.

## Data availability statement

The raw data supporting the conclusions of this article will be made available by the authors, without undue reservation.

## Ethics statement

The studies involving human participants were reviewed and approved by the Ethical Committee of the University Campus Bio-Medico of Rome. Written informed consent for participation was not required for this study in accordance with the national legislation and the institutional requirements.

## Author contributions

SS led the study design, data collection, data analysis, data interpretation, and manuscript writing. FM, GD’A, and DL assisted with data collection and analysis of the validation dataset. FM performed the statistical analysis. FS assisted the patients. MF, LL, DL, and GB assisted with computer queries, data analysis, and manuscript preparation. CB, AA, JM, and RM assisted with data collection and analysis of the development dataset as well as study design, data interpretation, and manuscript writing. CB, JM, AA, SC, and SA assisted with chart review, data analysis, and supervised all aspects of the investigation, as well as assisting with study design, data interpretation, and manuscript writing. All authors contributed to manuscript revision, read, and approved the submitted version.

## Abbreviations

ADM: adrenomedullin
ARDS: acute respiratory distress syndrome
AUC: areas under the curve
COVID-19: coronavirus disease 2019
CRP: C-reactive protein
MR-proADM: mid-regional-proAdrenomedullin
PCT: procalcitonin
ROC: receiver operating characteristic
SARS-CoV-2: severe acute respiratory distress syndrome coronavirus 2
SOFA: sequential organ failure assessment.
